# Supplementary material for: Counter culture: causes, extent and solutions of systematic bias in the analysis of behavioural counts
Source: PeerJ. 2023 Apr 4;11:e15059. doi: 10.7717/peerj.15059 (PMC10081455; doi:10.7717/peerj.15059)
Supplement: Supplemental Information 1 [file peerj-11-15059-s001.pdf]

Counter culture: Causes, extent and solutions of systematic bias in the analysis of behavioural counts

Supplementary Material

Joel L. Pick*, Nyil Khwaja, Michael A. Spence,

Malika Ihle & Shinichi Nakagawa

December 14, 2022

* Corresponding Author: joel.l.pick@gmail.com

Contents

S1 Refractory Period and Stochastic Error 3

S2 Demonstrating provisioning as a Poisson process using the data of Lendvai et al. 2015 6

S3 Studies Included in Literature Survey 9

S4 Simulations 24

S5 Calculating CV of expected rates 26

S6 Tutorial - Analysing Provisioning Data 28

S6.1 Exploring Provisioning Rate . . . 28

S6.2 Analysing Provisioning Rate as a Response Variable . . . 29

S6.2.1 Estimating repeatability with and without Poisson-distributed stochastic error . . . 29

S6.2.2 Estimating CV_B . . . 32

S6.2.3 Problems associated with analysing rate . . . 33

S6.2.4 Accounting for observation period . . . 34

S6.3 Analysing Provisioning Rate as a Predictor Variable . . . 36

S6.3.1 Accounting for observation period . . . 39

S1 Refractory Period and Stochastic Error

A Poisson process assumes an exponential distribution of inter-visit intervals (IVIs), which is a specific formulation of the gamma distribution. The gamma distribution can be described by two parameters α and β; in an exponential distribution the α parameter is fixed to 1 (Figure S1A). This results in a modal IVI of 0. When observing such a process for a certain observation period (t), the mean number of observations ($\bar{y}$) is calculated as

$$\bar{y} = \frac{t}{\mu} \tag{S1}$$

, where μ is the mean IVI. The stochastic error (σ_y) is

$$\sigma_y = \frac{t}{\alpha\mu} \tag{S2}$$

. Thus when $\alpha = 1$, as under a Poisson process, then the stochastic error is equal to the mean. This corresponds to a Poisson distribution of observations (Figure S1B).

In many scenarios it seems likely that the probability of very short IVIs would be low (i.e. modal IVI > 0), for example if there is a minimum realistic time in which a bird could find food. This can be described as there being a refractory period. Such distribution of IVIs would be described by a gamma distribution where $\alpha > 1$. Therefore, for the same μ (and $\bar{y}$), as α increases, the stochastic error decreases (Figure S1A). α describes the refractory period in terms of the expected interval length. When $\alpha = 2$ the refractory period is an average IVI long, and when $\alpha = 3$ the refractory period is 2 IVIs long, etc. The refractory period itself can be more intuitively thought of as the mode of the gamma distribution, $((\alpha - 1)/\beta)$, as this given a quantification in terms of a time period.

Using simulated data, we can demonstrate how stochastic error decreases with the presence of a refractory period, when the mean, and so observed number of visits, remains constant. We simulated visit data from a gamma distribution, with a mean IVI (μ) of

5 minutes, varying α. Assuming a total observation time (t) of 90 minutes, we counted all visits from intervals whose cumulative sum was less than or equal to 90, and repeated this 100 times to create 100 observations. Figure S1 shows the gamma distribution from which the intervals were simulated (Figure S1A) and the resulting the number of observations (Figure S1B). The mean number of observations is constant (here 18), regardless of the changing α, whilst the stochastic error decreases as a function of α (Figure S1D).

This process can be easily described with the Tweedie distribution, the variance of which is defined as

$$\sigma_y = \phi \bar{y}^{\xi} \tag{S3}$$

Therefore when $\xi = 1$ and $\phi = 1/\alpha$, the Tweedie distribution can be used to estimate the stochastic error. This is shown in Figure S1C.

For this to be used a population level α would have to be estimated and specified in the model. If the model was allowed to estimate ϕ, then it would include any residual or over-dispersion variance, and so would not be able to be used to estimate α.

In the main text, we mention that if the refractory period, and so α, is small then assuming Poisson distributed error results in less bias in effect size estimation than assuming no stochastic error. As the stochastic error is scaled by $1/\alpha$, we can see that when $1 \leq \alpha < 2$, the stochastic error will be closer to Poisson distributed error than zero, whilst if $\alpha > 2$ assuming no Poisson-distributed stochastic error would induce less bias than assuming Poisson-distributed stochastic error (although would still result in the underestimation of effect sizes). As can be seen from Figure S1A, when $\alpha > 2$ the refractory period is quite pronounced; this is not the case in distributions of inter-visit intervals from published studies (see text for references).

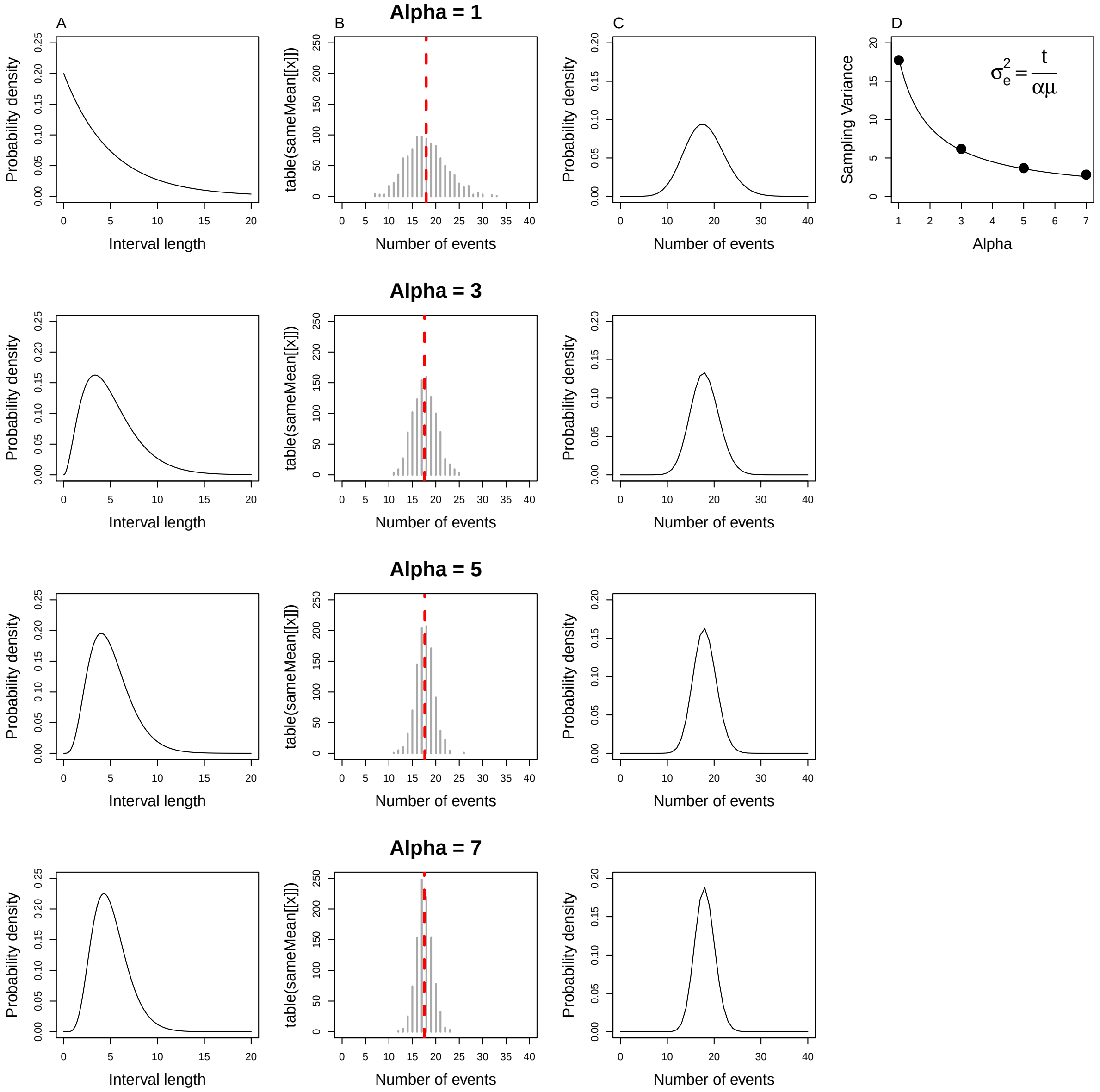

Figure S1: Effect of a refractory period on Poisson-distributed stochastic error. A) shows the gamma distributions from which inter-visit intervals were simulated, with varying values of alpha. B) shows the corresponding distributions of observation numbers from sampling these distributions over a 90 minute period. C) shows the probability density function from a Tweedie distribution that would best describe the distribution of counts arising from these gamma distributions of IVIs. D) shows the relationship between alpha and the resulting stochastic error; the points are from the simulated datasets, line represent theoretical expectations.

S2 Demonstrating provisioning as a Poisson process using the data of Lendvai et al. 2015

In a recent study, Lendvai *et al.* (2015) demonstrated that, as observation periods increase, the correlation between number of visits in the observations and in the whole day increases. We can use this data (Lendvai *et al.* 2015 Supplementary Materials) to further demonstrate how provisioning data matches the assumptions a Poisson process. Lendvai *et al.* (2015) measured provisioning over the course of a whole day, broken up into the number of visits per 15 minute period. We subset their data to include records from 10:30 to 20:45; we discarded data before this time period, as there was a high proportion of individuals with missing data, and after this time as most individuals had stopped provisioning (Fig S2A and B). This left us with 41, 15 minute observation periods, of 128 individuals.

With this data set we combined adjacent 15 minute time blocks to create new, longer, observation periods, i.e. to make 30 minute observations, we combined pairs of adjacent time blocks to make 20 new 30 minute time blocks. We did this to create observation periods increasing in length by 15 minutes, up to 615 minutes (i.e. 41 *15 minutes). Depending on the length of the observation period, we could not use all the time blocks (i.e. if the length of the new observation period did not divide exactly into 615 minutes). We therefore preferentially took observation from the latter part the day, as there were less missing values here (see Figure S2C).

To calculate the observed coefficient of variation (CV = $\sigma_y/\bar{y}$), for each observation period we pooled the new data and calculated CV across this data. Using the observed CV (CV_{obs}) and mean ($\bar{y}$) across different observation periods, we estimated the expected CV

(CV_{exp}) using a nonlinear model with the `nls` function in *R* with the form

$$CV_{obs} = \frac{\sqrt{\bar{y} + (\bar{y}CV_{exp})^2}}{\bar{y}} \tag{S4}$$

The predictions from this model are plotted in Figure 2B.

To calculate repeatability, we use these new observation periods as repeated measures, and calculated repeatability across the new data, in a linear mixed model, with individual ID as a random effect, using the *lme4* package in *R* (Bates *et al.*, 2015). Repeatability was then calculated as individual variance / (individual variance + residual variance). This was only possible for observation periods up to 300 mins, as at least two time blocks were needed to estimate repeatability. Using the observed repeatability (ICC_{obs}), mean ($\bar{y}$) across different observation periods and the expected CV (CV_{exp}) as calculated above, we estimated the true repeatability (ICC) using a nonlinear model with the form

$$ICC_{obs} = \frac{ICC\bar{y}CV_{exp}^2}{1 + \bar{y}CV_{exp}^2} \tag{S5}$$

The predictions from this model are plotted in Figure 2D.

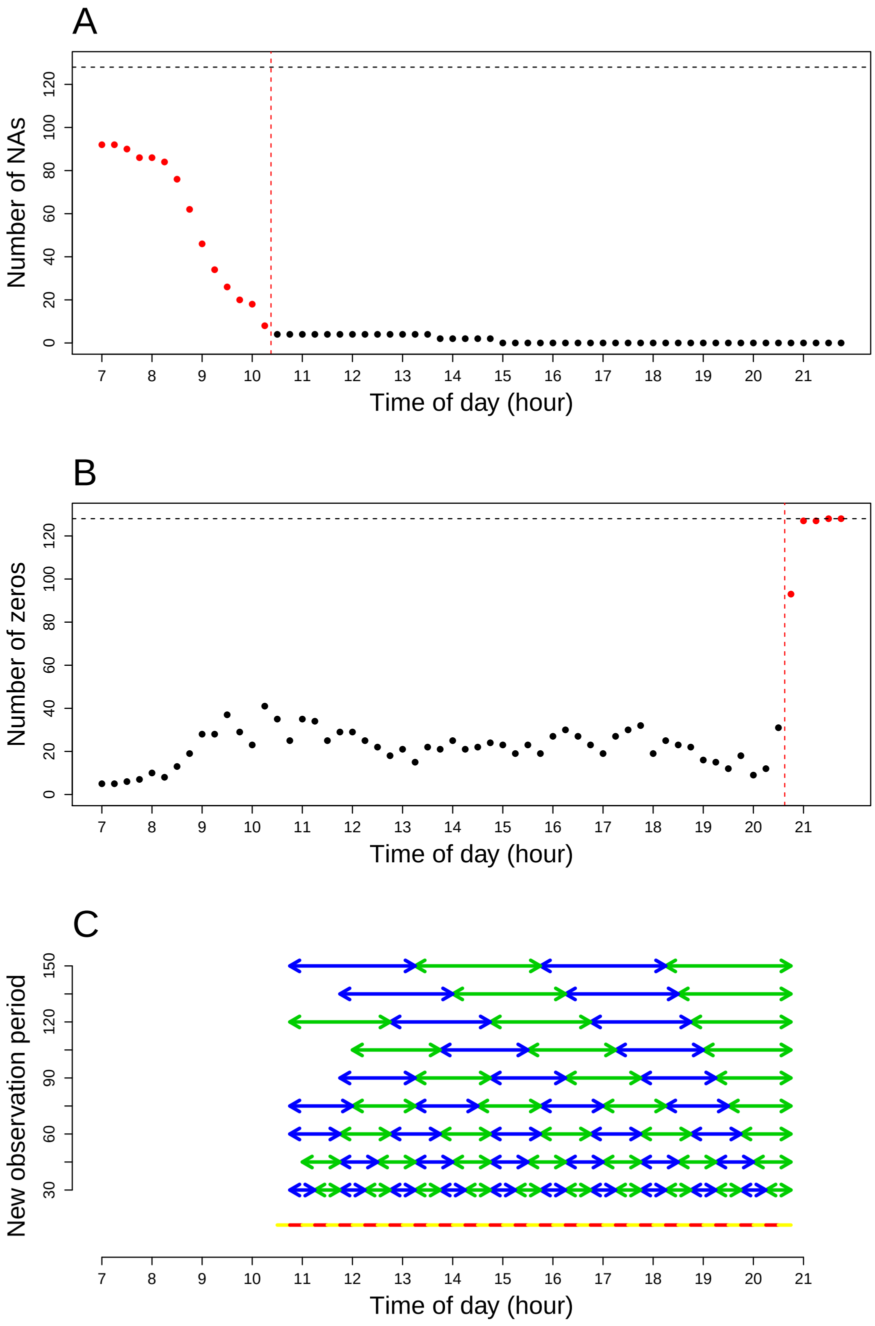

Figure S2: Selection and manipulation of data from Lendvai et al. 2015. A) and B) show time periods that were excluded because of A) high level of missing data and B) high level of individuals that had stopped provisioning. Red points are those that were excluded. Black dotted line shows total number of individuals. C) shows how new observation periods were made; the red and yellow line shows that 15 minute observation periods, and the blue and green line show how there were combined to make longer observation periods.

S3 Studies Included in Literature Survey

The reference numbers refer to the study reference in table S1.

5. Blas, J., Abaurrea, T., D'Amico, M., Barcellona, F., Revilla, E., Roman, J. & Carrete, M. (2016) Management-related traffic as a stressor eliciting parental care in a roadside-nesting bird: the european bee-eater *Merops apiaster*. *Plos One*, **11**, e0164371.

10. Young, R.C., Barger, C.P., Dorresteijn, I., Haussmann, M.F. & Kitaysky, A.S. (2016) Telomere length and environmental conditions predict stress levels but not parental investment in a long-lived seabird. *Marine Ecology Progress Series*, **556**, 251-259.

11. Barger, C.P., Young, R.C., Will, A., Ito, M. & Kitaysky, A.S. (2016) Resource partitioning between sympatric seabird species increases during chick-rearing. *Ecosphere*, **7**, e01447.

19. Auer, S.A., Islam, K., Wagner, J.R., Summerville, K.S. & Barnes, K.W. (2016) The diet of cerulean warbler (*Setophaga cerulea*) nestlings and adult nest provisioning behaviors in southern indiana. *Wilson Journal Of Ornithology*, **128**, 573-583.

22. Amininasab, S.M., Kingma, S.A., Birker, M., Hildenbrandt, H. & Komdeur, J. (2016) The effect of ambient temperature, habitat quality and individual age on incubation behaviour and incubation feeding in a socially monogamous songbird. *Behavioral Ecology And Sociobiology*, **70**, 1591-1600.

24. Koenig, W.D. & Walters, E.L. (2016) Provisioning patterns in the cooperatively breeding acorn woodpecker: does feeding behaviour serve as a signal? *Animal Behaviour*, **119**, 125-134.

27. Cantarero, A., Lopez-Arrabe, J., Plaza, M., Saavedra-Garces, I. & Moreno, J.

(2016) Males feed their mates more and take more risks for nestlings with larger female-built nests: an experimental study in the nuthatch *Sitta europaea. Behavioral Ecology And Sociobiology*, **70**, 1141-1150.

28. Schroeder, J., Hsu, Y.H., Winney, I., Simons, M., Nakagawa, S. & Burke, T. (2016) Predictably philandering females prompt poor paternal provisioning. *American Naturalist*, **188**, 219-230.

29. Tanaka, K.D., Morimoto, G. & Ueda, K. (2016) Equivocal responses of feeding parents to experimental brood sizes in a hawk-cuckoo host: brood size as a reference for parental provisioning decisions? *Ethology*, **122**, 640-648.

33. Ren, Q.M., Luo, S., Du, X.J., Chen, G.L., Song, S. & Du, B. (2016) Helper effects in the azure-winged magpie cyanopica cyana in relation to highly-clumped nesting pattern and high frequency of conspecific nest-raiding. *Journal Of Avian Biology*, **47**, 449-456.

35. Scioscia, G., Rey, A.R. & Schiavini, A. (2016) Breeding biology of magellanic penguins (*Spheniscus magellanicus*) at the beagle channel: interannual variation and its relationship with foraging behaviour. *Journal Of Ornithology*, **157**, 773-785.

37. Romano, A., Bazzi, G., Caprioli, M., Corti, M., Costanzo, A., Rubolini, D. & Saino, N. (2016) Nestling sex and plumage color predict food allocation by barn swallow parents. *Behavioral Ecology*, **27**, 1198-1205.

38. Liebl, A.L., Browning, L.E. & Russell, A.F. (2016) Manipulating carer number versus brood size: complementary but not equivalent ways of quantifying carer effects on offspring. *Behavioral Ecology*, **27**, 1247-1254.

40. Endo, S. & Ueda, K. (2016) Factors affecting female incubation behavior in the bullheaded shrike. *Ornithological Science*, **15**, 151-161.

42. Dzielski, S.A., Van Doren, B.M., Hruska, J.P. & Hite, J.M. (2016) Reproductive

biology of the Sapayoa (*Sapayoa aenigma*), the "old world suboscine" of the new world. *Auk*, **133**, 347-363.

43. Carla, M.R. & Fontana, C.S. (2016) Breeding biology of the tropeiro seedeater (*Sporophila beltoni*). *Auk*, **133**, 484-496.

54. Yasukawa, K., Berrios, H.K. & Johannes, A.W. (2016) A test of the nestling discrimination hypothesis for parasitism of red-winged blackbirds (*Agelaius phoeniceus*) by brown-headed cowbirds (molothrus ater). *Wilson Journal Of Ornithology*, **128**, 437-441.

55. Hanson, M.R. & Baldwin, J.D. (2016) Provisioning rates suggest food limitation for breeding bald eagles in their southernmost range. *Southeastern Naturalist*, **15**, 365-381.

67. Lucass, C., Eens, M. & Muller, W. (2016) When ambient noise impairs parent-offspring communication. *Environmental Pollution*, **212**, 592-597.

69. Lucass, C., Iserbyt, A., Eens, M. & Muller, W. (2016) Structural (uv) and carotenoid-based plumage coloration signals for parental investment? *Ecology And Evolution*, **6**, 3269-3279.

70. Liebl, A.L., Nomano, F.Y., Browning, L.E. & Russell, A.F. (2016) Experimental evidence for fully additive care among male carers in the cooperatively breeding chestnut-crowned babbler. *Animal Behaviour*, **115**, 47-53.

73. Cornell, A. & Williams, T.D. (2016) Individual quality and double-brooding in a highly synchronous songbird population. *Auk*, **133**, 251-260.

75. Potticary, A.L., Dowling, J.L., Barron, D.G., Baldassarre, D.T. & Webster, M.S. (2016) Subtle benefits of cooperation to breeding males of the red-backed fairywren. *Auk*, **133**, 286-297.

76. Burtka, J.L., Lovern, M.B. & Grindstaff, J.L. (2016) Baseline hormone levels are linked to reproductive success but not parental care behaviors. *General And Comparative Endocrinology*, **229**, 92-99.

85. Pretelli, M.G., Isacch, J.P. & Cardoni, D.A. (2016) Variation in parental care in the spectacled tyrant *Hymenops perspicillatus* is associated with increased nest predation in grassland fragments. *Journal Of Ornithology*, **157**, 451-460.

86. Morrison, K.W., Morrison, N.C., Buchheit, R.M., Dunn, R., Battley, P.F. & Thompson, D.R. (2016) The canalized parental roles of a Eudyptes penguin constrain provisioning and growth of chicks during nutritional stress. *Behavioral Ecology And Sociobiology*, **70**, 467-479.

87. Moks, K., Tilgar, V., Thomson, R.L., Calhim, S., Jarvisto, P.E., Schuett, W., Velmala, W. & Laaksonen, T. (2016) Predator encounters have spatially extensive impacts on parental behaviour in a breeding bird community. *Proceedings Of The Royal Society B-biological Sciences*, **283**, 20160020.

92. Ostreiher, R. & Heifetz, A. (2016) The blessing of having younger nestmates: the case of the arabian babbler. *Behavioral Ecology*, **27**, 393-400.

94. Bebbington, K. & Hatchwell, B.J. (2016) Coordinated parental provisioning is related to feeding rate and reproductive success in a songbird. *Behavioral Ecology*, **27**, 652-659.

96. Heist, C.A. & Ritchison, G. (2016) Effects of variation in nestling hunger levels and begging on the provisioning behavior of male and female eastern phoebes (*Sayornis phoebe*). *Wilson Journal Of Ornithology*, **128**, 132-143.

97. Botero-Delgadillo, E. & Vasquez, R.A. (2016) Nest architecture, clutch size, nestling growth patterns and nestling attendance of the fire-eyed diucon (*Xolmis pyrope*) in

north-central chile. *Wilson Journal Of Ornithology*, **128**, 184-190.

100. Michalczuk, J. & Michalczuk, M. (2016) Differences in reproductive investment between male and female syrian woodpeckers *Dendrocopos syriacus* (hemprich and ehrenberg, 1833) in a newly-colonised area. *Acta Zoologica Bulgarica*, **68**, 77-84.

101. Fronstin, R.B., Christians, J.K. & Williams, T.D. (2016) Experimental reduction of haematocrit affects reproductive performance in european starlings. *Functional Ecology*, **30**, 398-409.

102. Sanchez-Martinez, M.A. & Londono, G.A. (2016) Nesting behavior of male and female undulated antshrikes (*Frederickena unduliger*). *Journal Of Field Ornithology*, **87**, 21-28.

105. Mainwaring, M.C. & Hartley, I.R. (2016) Experimentally reduced nest-building costs result in higher provisioning rates but not increased offspring fitness in blue tits. *Avian Biology Research*, **9**, 52-57.

114. Lucass, C., Korsten, P., Eens, M. & Muller, W. (2016) Within-family parent-offspring co-adaptation in a wild bird: on static traits, behavioural reaction norms, and sex differences. *Functional Ecology*, **30**, 274-282.

120. Robertson, G.S., Bolton, M. & Monaghan, P. (2016) Influence of diet and foraging strategy on reproductive success in two morphologically similar sympatric seabirds. *Bird Study*, **63**, 319-329.

124. Chutter, C.M., Redmond, L.J., Cooper, N.W., Dolan, A.C., Duffield, D. & Murphy, M.T. (2016) Paternal behaviour in a socially monogamous but sexually promiscuous Passerine bird. *Behaviour*, **153**, 443-466.

126. Bueno-Enciso, J., Ferrer, E.S., Barrientos, R., Serrano-Davies, E. & Sanz, J.J. (2016) Habitat fragmentation influences nestling growth in mediterranean blue and

great tits. *Acta Oecologica-international Journal Of Ecology*, **70**, 129-137.

131. Kadin, M., Olsson, O., Hentati-Sundberg, J., Ehrning, E.W. & Blenckner, T. (2016) Common guillemot *Uria aalge* parents adjust provisioning rates to compensate for low food quality. *Ibis*, **158**, 167-178.

136. McDonald, P.G., Rollins, L.A. & Godfrey, S. (2016) The relative importance of spatial proximity, kin selection and potential 'greenbeard' signals on provisioning behaviour among helpers in a cooperative bird. *Behavioral Ecology And Sociobiology*, **70**, 133-143.

137. Dakin, R., Lendvai, A.Z., Ouyang, J.Q., Moore, I.T. & Bonier, F. (2016) Plumage colour is associated with partner parental care in mutually ornamented tree swallows. *Animal Behaviour*, **111**, 111-118.

139. Exposito-Granados, M., Parejo, D. & Aviles, J.M. (2016) Sex-specific parental care in response to predation risk in the european roller, *Coracias garrulus*. *Ethology*, **122**, 72-79.

148. Du, B., Guan, M.M., Ren, Q.M. & Chen, G.L. (2015) Cuckolded male ground tits increase parental care for the brood. *Animal Behaviour*, **110**, 61-67.

150. Wetzel, D.P., Hatch, M.I. & Westneat, D.F. (2015) Genetic sources of individual variation in parental care behavior. *Behavioral Ecology And Sociobiology*, **69**, 1933-1943.

151. Cram, D.L., Blount, J.D. & Young, A.J. (2015) The oxidative costs of reproduction are group-size dependent in a wild cooperative breeder. *Proceedings Of The Royal Society B-biological Sciences*, **282**, 20152031.

153. Lendvai, A.Z., Akcay, C., Ouyang, J.Q., Dakin, R., Domalik, A.D., St John, P.S., Stanback, M., Moore, I.T. & Bonier, F. (2015) Analysis of the optimal duration of behavioral observations based on an automated continuous monitoring system in tree

swallows (*Tachycineta bicolor*): is one hour good enough? *Plos One*, **10**, e0141194.

160. Leonard, M.L., Horn, A.G., Oswald, K.N. & McIntyre, E. (2015) Effect of ambient noise on parent-offspring interactions in tree swallows. *Animal Behaviour*, **109**, 1-7.

163. Ouyang, J.Q., Lendvai, A.Z., Dakin, R., Domalik, A.D., Fasanello, V.J., Vassallo, B.G., Haussmann, M.F., Moore, I.T. & Bonier, F. (2015) Weathering the storm: parental effort and experimental manipulation of stress hormones predict brood survival. *Bmc Evolutionary Biology*, **15**, 219.

164. Dunn, J.C., Hamer, K.C. & Benton, T.G. (2015) Anthropogenically-mediated density dependence in a declining farmland bird. *Plos One*, **10**, e0139492.

166. Laubach, Z.M., Perng, W., Lombardo, M., Murdock, C. & Foufopoulos, J. (2015) Determinants of parental care in mountain white-crowned sparrows (*Zonotrichia leucophrys* oriantha). *Auk*, **132**, 893-902.

172. Wiebe, K.L. & Slagsvold, T. (2015) Foraging trade-offs between prey size, delivery rate and prey type: how does niche breadth and early learning of the foraging niche affect food delivery? *Ethology*, **121**, 1010-1017.

174. Llambias, P.E., Carro, M.E. & Fernandez, G.J. (2015) Latitudinal differences in life-history traits and parental care in northern and southern temperate zone house wrens. *Journal Of Ornithology*, **156**, 933-942.

178. Rouaiguia, M., Lahlah, N., Bensaci, E. & Houhamdi, M. (2015) Feeding behaviour and the role of insects in the diet of northern house-martin (*Delichon urbica* meridionalis) nestlings in northeastern algeria. *African Entomology*, **23**, 329-341.

181. Chiarani, E. & Fontana, C.S. (2015) Breeding biology of the lesser grass-finch (*Emberizoides ypiranganus*) in southern brazilian upland grasslands. *Wilson Journal Of Ornithology*, **127**, 441-456.

184. Fowler, M.A. & Williams, T.D. (2015) Individual variation in parental workload and breeding productivity in female european starlings: is the effort worth it? *Ecology And Evolution*, **5**, 3585-3599.

190. Yoon, J., Ha, H.S., Jung, J.S. & Park, S.R. (2015) Post-mating sexual behaviors of oriental storks (*Ciconia boyciana*) in captivity. *Zoological Science*, **32**, 331-335.

191. Riehl, C. & Strong, M.J. (2015) Social living without kin discrimination: experimental evidence from a communally breeding bird. *Behavioral Ecology And Sociobiology*, **69**, 1293-1299.

194. Adams, M.J., Robinson, M.R., Mannarelli, M.E. & Hatchwell, B.J. (2015) Social genetic and social environment effects on parental and helper care in a cooperatively breeding bird. *Proceedings Of The Royal Society B-biological Sciences*, **282**, 20150689.

195. Robertson, G.S., Bolton, M. & Monaghan, P. (2015) Parental resource allocation among offspring varies with increasing brood age in black-legged kittiwakes *Rissa tridactyla*. *Bird Study*, **62**, 303-314.

196. Whytock, R.C., Davis, D., Whytock, R.T., Burgess, M.D., Minderman, J. & Mallord, J.W. (2015) Wood warbler *Phylloscopus sibilatrix* nest provisioning rates are correlated with seasonal caterpillar availability in british oak quercus woodlands. *Bird Study*, **62**, 339-347.

200. Nomano, F.Y., Browning, L.E., Savage, J.L., Rollins, L.A., Griffith, S.C. & Russell, A.F. (2015) Unrelated helpers neither signal contributions nor suffer retribution in chestnut-crowed babblers. *Behavioral Ecology*, **26**, 986-995.

201. Grunst, A.S. & Grunst, M.L. (2015) Context-dependent relationships between multiple sexual pigments and paternal effort. *Behavioral Ecology*, **26**, 1170-1179.

213. Rishworth, G.M. & Pistorius, P.A. (2015) Protection and provisioning: the role of

parental behaviour in terms of chick growth and survival in a pelagic seabird. *Marine Ecology Progress Series*, **530**, 153-162.

215. Auclair, M.M.M., Ono, K.A. & Perlut, N.G. (2015) Brood provisioning and nest survival of *Ardea herodias* (great blue heron) in maine. *Northeastern Naturalist*, **22**, 307-317.

223. Welch, B.C. & Boal, C.W. (2015) Prey use and provisioning rates of urban-nesting mississippi kites in west texas. *Journal Of Raptor Research*, **49**, 141-151.

224. Bowers, E.K., Bowden, R.M., Sakaluk, S.K. & Thompson, C.F. (2015) Immune activation generates corticosterone-mediated terminal reproductive investment in a wild bird. *American Naturalist*, **185**, 769-783.

227. Bolopo, D., Canestrari, D., Roldan, M., Baglione, V. & Soler, M. (2015) High begging intensity of great spotted cuckoo nestlings favours larger-size crow nest mates. *Behavioral Ecology And Sociobiology*, **69**, 873-882.

233. Wegmann, M., Voegeli, B. & Richner, H. (2015) Oxidative status and reproductive effort of great tits in a handicapping experiment. *Behavioral Ecology*, **26**, 747-754.

245. Du, B., Liu, C.J. & Bao, S.J. (2015) Begging form and growth pattern of nestlings correlate with parental food-allocation patterns in the horned lark (*Eremophila alpestris*). *Canadian Journal Of Zoology*, **93**, 273-279.

246. Diniz, P., Ramos, D.M. & Macedo, R.H. (2015) Attractive males are less than adequate dads in a multimodal signalling Passerine. *Animal Behaviour*, **102**, 109-117.

253. Wegmann, M., Voegeli, B. & Richner, H. (2015) Physiological responses to increased brood size and ectoparasite infestation: adult great tits favour self-maintenance. *Physiology & Behavior*, **141**, 127-134.

254. Montoya, B. & Torres, R. (2015) Male skin color signals direct and indirect benefits in a species with biparental care. *Behavioral Ecology*, **26**, 425-434.

262. Mullers, R.H.E. & Amar, A. (2015) Parental nesting behavior, chick growth and breeding success of shoebills (*Balaeniceps rex*) in the bangweulu wetlands, zambia. *Waterbirds*, **38**, 1-9.

274. Bodrati, A., Cockle, K.L., Di Sallo, F.G., Ferreyra, C., Salvador, S.A. & Lammertink, M. (2015) Nesting and social roosting of the ochre-collared piculet (*Picumnus temminckii*) and white-barred piculet (Picumnus cirratus), and implications for the evolution of woodpecker (picidae) breeding biology. *Ornitologia Neotropical*, **26**, 223-244.

278. Jimeno, B. & Gil, D. (2015) Parent-absent calls are related to nestling reaction time and parental food allocation in the spotless starling. *Behaviour*, **152**, 1413-1431.

279. Goyert, H.F. (2015) Foraging specificity and prey utilization: evaluating social and memory-based strategies in seabirds. *Behaviour*, **152**, 861-895.

281. Oberg, M., Arlt, D., Part, T., Laugen, A.T., Eggers, S. & Low, M. (2015) Rainfall during parental care reduces reproductive and survival components of fitness in a Passerine bird. *Ecology And Evolution*, **5**, 345-356.

282. Grunst, A.S., Grunst, M.L. & Rotenberry, J.T. (2015) Sexual pigmentation and parental risk-taking in yellow warblers *Setophaga petechia*. *Journal Of Avian Biology*, **46**, 9-17.

283. Schneider, N.A. & Griesser, M. (2015) Within-season increase in parental investment in a long-lived bird species: investment shifts to maximize successful reproduction? *Journal Of Evolutionary Biology*, **28**, 231-240.

288. Garcia-Navas, V., Valera, F. & Griggio, M. (2015) Nest decorations: an 'extended'

female badge of status? *Animal Behaviour*, **99**, 95-107.

2022-3. Ritchison, G., Lewis, L. & Heist, C.A. (2022) Provisioning behavior of male and female Loggerhead Shrikes. *Avian Biology Research*, **15**, 93-99. http://dx.doi.org/10.1177/17581559221092692

2022-4. Eguchi, K., Mikami, K., Yamaguchi, N. & Noske, R.A. (2022) Inter-sexual differences in contributions of helpers in a tropical population of the cooperatively breeding Grey-crowned Babbler Pomatostomus temporalis. *Emu-austral Ornithology*, **122**, 77-86. http://dx.doi.org/10.1080/01584197.2022.2072347

2022-5. Tilgar, V., Hein, K. & Viigipuu, R. (2022) Anthropogenic noise alters the perception of a predator in a local community of great tits. *Animal Behaviour*, **189**, 91-99. http://dx.doi.org/10.1016/j.anbehav.2022.05.001

2022-6. Lopez-Lopez, P., Perona, A.M., Egea-Casas, O., Morant, J. & Urios, V. (2022) Tri-axial accelerometry shows differences in energy expenditure and parental effort throughout the breeding season in long-lived raptors. *Current Zoology*, **68**, 57-67. http://dx.doi.org/10.1093/cz/zoab010

2022-7. Enns, J. & Williams, T.D. (2022) Paying attention but not coordinating: parental care in European starlings, Sturnus vulgaris. *Animal Behaviour*, **193**, 113-124. http://dx.doi.org/10.1016/j.anbehav.2022.09.009

2022-10. Walter, L.A., Viverette, C. & Bulluck, L. (2022) Parental care in a sexually monomorphic Picid, the Red-headed Woodpecker. *Journal Of Field Ornithology*, **93**, 3. http://dx.doi.org/10.5751/JFO-00089-930203

2022-11. White, A.F. & Dawson, R.D. (2022) Manipulations of brood age reveal limited parental flexibility in an insectivorous passerine. *Animal Behaviour*, **184**, 99-109. http://dx.doi.org/10.1016/j.anbehav.2021.12.006

2022-12. Baldan, D. & van Loon, E.E. (2022) Songbird parents coordinate offspring provisioning at fine spatio-temporal scales. *Journal Of Animal Ecology*, **91**, 1316-1326. http://dx.doi.org/10.1111/1365-2656.13702

2022-13. Sandvig, E.M., Coulson, T., Robertson, B.C., Feeney, W.E. & Clegg, S.M. (2022) Insular nestling growth and its relationship to parental care effort in Silvereyes, Zosterops lateralis. *Emu-austral Ornithology*, **122**, 193-202. http://dx.doi.org/10.1080/01584197.2022.2105723

2022-15. Arrieta, R.S., Campagna, L., Mahler, B. & Llambias, P.E. (2022) Neither paternity loss nor perceived threat of cuckoldry affects male nestling provisioning in grass wrens. *Behavioral Ecology And Sociobiology*, **76**, 147. http://dx.doi.org/10.1007/s00265-022-03253-y

2022-19. Lukhele, S.M., Widdows, C.D. & Kirschel, A.N.G. (2022) Nest provisioning and diet of nestling yellow-rumped tinkerbird Pogoniulus bilineatus. *African Journal Of Ecology*, **60**, 75-78. http://dx.doi.org/10.1111/aje.12927

2022-24. Morvai, B., Fazekas, E.A., Miklosi, A. & Pogany, A. (2022) Genetic and Social Transmission of Parental Sex Roles in Zebra Finch Families. *Frontiers In Ecology And Evolution*, **9**, 799996. http://dx.doi.org/10.3389/fevo.2021.799996

2022-29. Dharmasiri, M.E., Barber, C.A. & Horn, A.G. (2022) Nestling European Starlings (Sturnus vulgaris) adjust their begging calls in noise. *Bioacoustics-the International Journal Of Animal Sound And Its Recording*, **31**, 594-613. http://dx.doi.org/10.1080/09524622.2021.2008495

2022-30. Madden, S.A., McDermott, M.T. & Safran, R.J. (2022) Visitation rate, but not foraging range, responds to brood size manipulation in an aerial insectivore. *Behavioral Ecology And Sociobiology*, **76**, 139. http://dx.doi.org/10.1007/s00265-022-03244-z

2022-34. Velasco, A.C., Ferrer, E.S. & Sanz, J.J. (2022) Exploratory behaviour and foraging strategies in Mediterranean blue tits. *Animal Biodiversity And Conservation*, **45**, 287-+. http://dx.doi.org/10.32800/abc.2022.45.0287

2022-44. Hagler, S.J., Gibbons, A., Bednarz, J.C., Clark, W.S. & Bonter, D.N. (2022) NEST PROVISIONING AND SOCIALITY AT HARRIS'S HAWK NESTS IN SOUTH TEXAS. *Journal Of Raptor Research*, **56**, 161-170. http://dx.doi.org/10.3356/JRR-21-39

2022-49. Vali, U. & Magalhaes, A. (2022) Web-based citizen science as a tool in conservation research: A case study of prey delivery by the Lesser Spotted Eagle. *Plos One*, **17**, e0261655. http://dx.doi.org/10.1371/journal.pone.0261655

2022-60. Liu, Q.X., Gelok, E., Fontein, K., Slabbekoorn, H. & Riebel, K. (2022) An experimental test of chronic traffic noise exposure on parental behaviour and reproduction in zebra finches. *Biology Open*, **11**, bio059183. http://dx.doi.org/10.1242/bio.059183

2022-66. Halliwell, C., Beckerman, A.P., Germain, M., Patrick, S.C., Leedale, A.E. & Hatchwell, B.J. (2022) Coordination of care by breeders and helpers in the cooperatively breeding long-tailed tit. *Behavioral Ecology*, **33**, 844-858. http://dx.doi.org/10.1093/beheco/arac048

2022-70. Garrett, D.R., Pelletier, F., Garant, D. & Belisle, M. (2022) Negative effects of agricultural intensification on the food provisioning rate of a declining aerial insectivore. *Ecosphere*, **13**, e4227. http://dx.doi.org/10.1002/ecs2.4227

2022-74. Soler, M., Ruiz-Raya, F., Sanchez-Perez, L., Ibanez-Alamo, J.D. & Soler, J.J. (2022) Functional explanation of extreme hatching asynchrony: Male Manipulation Hypothesis. *Zoological Research*, **43**, 843-850. http://dx.doi.org/10.24272/j.issn.2095-8137.2021.455

2022-86. Shiao, M.T., Yao, M.C. & Chuang, M.C. (2022) Provisioning flexibility and nestling growth of Green-backed Tit Parus monticolus in a mosaic of mixed-oak forest and cedar plantation in Taiwan. *Ornithological Science*, **21**, 165-176.

2022-92. Coon, J.J., Nelson, S.B.M., Bradley, I.A., Rola, K.E. & Miller, J.R. (2022) Increased abundance and productivity of a grassland bird after experimental control of invasive tall fescue. *Restoration Ecology*, **30**, e13709. http://dx.doi.org/10.1111/rec.13709

2022-96. Murakami, R., Sawada, A., Ono, H. & Takagi, M. (2022) The effect of experience on parental role division in Ryukyu Scops Owl Otus elegans. *Ornithological Science*, **21**, 3-2-44.

2022-98. Gao, Y.L., Xiao, H.Q., Han, H., Wei, W., Zhou, H. & Zhang, Z.J. (2022) Comparison of breeding strategies of two sympatric thrush species in an alpine environment. *Frontiers In Ecology And Evolution*, **10**, 1049983. http://dx.doi.org/10.3389/fevo.2022.1049983

2022-106. Aviles, J.M., Cruz-Miralles, A. & Parejo, D. (2022) Moonlight influence on quality of nestlings of scops owls depends on paternal coloration. *Behavioral Ecology*, **33**, 376-385. http://dx.doi.org/10.1093/beheco/arab139

2022-133. Taff, C.C., Johnson, B.A., Anker, A.T., Rodriguez, A.M., Houtz, J.L., Uehling, J.J. & Vitousek, M.N. (2022) No apparent trade-off between the quality of nest-grown feathers and time spent in the nest in an aerial insectivore, the Tree Swallow. *Ornithology*, **139**. http://dx.doi.org/10.1093/ornithology/ukac017

2022-136. Lane, S.J. & Sewall, K.B. (2022) What about Females? Urban Female Song Sparrows Elevate Aggressive Signaling Compared to Rural. *Integrative And Comparative Biology*. http://dx.doi.org/10.1093/icb/icac106

2022-144. Cousseau, L., Van de Loock, D., Apfelbeck, B., Githiru, M., Matthysen, E. & Lens, L. (2022) Kin do not always help: testing multiple hypotheses on nest feeding in a cooperatively breeding bird. *Behavioral Ecology.* http://dx.doi.org/10.1093/beheco/arac073

S4 Simulations

We ran simulations to demonstrate the difference between analysing provisioning rate when correcting and not correcting for Poisson-distributed stochastic error, with provisioning rate as both response and predictor variables, across different mean number of observed visits. For each mean, we simulated a dataset of 100 observations of provisioning rate with an expected CV of 0.3. For provisioning as a response variable, we simulated provisioning rate being affected by a single variable, which accounted to 50% of variation in provisioning rate on the expected scale ($R^2 = 0.5$). We ran two models; a linear model and a GLMM with Poisson error distribution and observation level random effect, the latter using the *lme4* package in *R* (Bates *et al.*, 2015). Both models included the observed number of visits as a response variable. For provisioning as a predictor variable, we simulated provisioning rate explaining 50% of variation in a another variable, on the expected scale ($R^2 = 0.5$). Again we ran two models; a linear model and a measurement error model, the latter using *Stan* (Carpenter *et al.*, 2017). Both models had number of visits as a predictor variable, but the measurement error model accounted for the uncertainty in observed number of visits. In both cases R^2 was calculated as

$$\frac{\sigma_f^2}{\sigma_f^2 + \sigma_r^2} \tag{S6}$$

where σ_f^2 is the variance due to the fixed effects calculated following Nakagawa & Schielzeth (2013) and σ_r^2 is the residual variance, which in the GLMM was the variance associated with the observation level random effect. All code for these simulations can be found at https://doi.org/10.5281/zenodo.7439115.

In order to explore the conditions under which we might see increased bias and lower precision, we again simulated provisioning rate as a response variable, being affected by a single variable, with an R^2 of 0.5 (θ). In these simulations we also varied the expected CV. For each expected CV and mean combination we simulated 1000 datasets, of 100

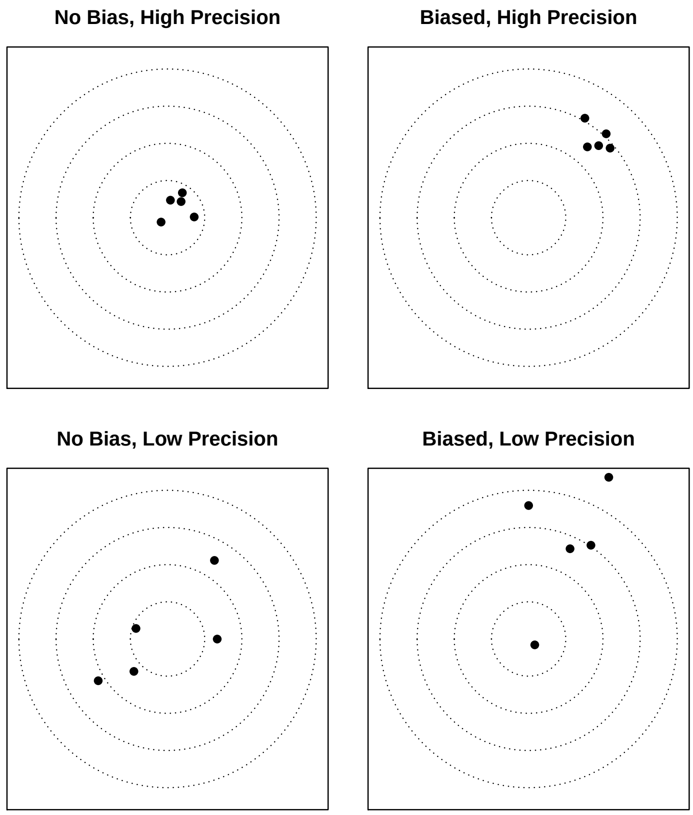

Figure S3: Bias and Precision

observations. For each dataset, we ran a GLMM with Poisson error distribution and observation level random effect, and estimated R2 ($\hat{\theta}$). Bias was calculated as $\theta - E(\hat{\theta})$, precision as $1/\sigma_{\hat{\theta}}$. Bias is measure of whether, and how much, estimates differ from the true value, whilst precision is a measure of variability in the estimates. This is shown graphically in Figure S3

For these latter simulations, we also calculated expected CV from the simulated datasets, following the equations in Box 2 to explore the conditions under which expected CV cannot be calculated, shown in Figure II.

S5 Calculating CV of expected rates

To calculate the expected CV, we can use the mean ($\bar{y}$) and variance of the observed number of arrivals (σ_y^2) in a population:

$$CV_{exp} = \frac{\sqrt{\sigma_y^2 - \bar{y}}}{\bar{y}} \tag{S7}$$

This formula works specifically for observations of the same length. When observation periods differ this can be accounted for by removing the variation due to observation period (t)

$$\hat{y} = y\bar{t}/t \tag{S8}$$

where $\hat{y}$ is the adjusted arrival rate. Here we have derived a rate, but importantly we have kept the mean number of visits the same. $\hat{y}$ can then be substituted into equation 3, in place of y.

Once the number of observed arrivals are transformed to a different scale (e.g. the number of arrivals is standardised to arrivals/hour) equation S7 is no longer valid, as Poisson distributed stochastic error does not scale in the same way as the expected variance in arrival rates. Therefore, the mean no longer represents the stochastic variance. Imagine a 120 min observation period with a mean of 20 ± 8 arrivals; the expected CV would be calculated as 0.332. However, when transformed to arrivals/hour, the mean±SD becomes 10 ± 4 arrivals/hour, and expected CV is calculated as 0.245, as we are overestimating the amount of Poisson sampling error. In order to estimate the true expected CV, the mean and SD of number of observed arrivals should therefore be back transformed to the scale on which the data was collected (i.e. by multiplying by the mean observation period; $\bar{t}$). This allows us to see why using rates instead of observed counts in a Poisson models results in an under- or overestimation of effect size. The model estimates the stochastic error based on the counts it is presented with. When the scale is changed, the model does

this incorrectly - if the counts are smaller than those observed, as in the example above, the stochastic error is overestimated, and so are effect sizes (Supplementary Material S6).

It is also important to note that when the mean number of observed visits is low, the estimation of expected CV may not be possible. As the variance is so dominated by stochastic error, random fluctuations in this error (induced by sampling error) can lead to the mean being larger than the variance meaning that $\sigma_y^2 - \bar{y}$ is negative. We can see this pattern in both simulated data, and in data from the literature review (Figure S4). Moreover, this is likely why we see an upward bias in effect size occur in the Poisson models, as the residual (i.e. expected) variance is underestimated.

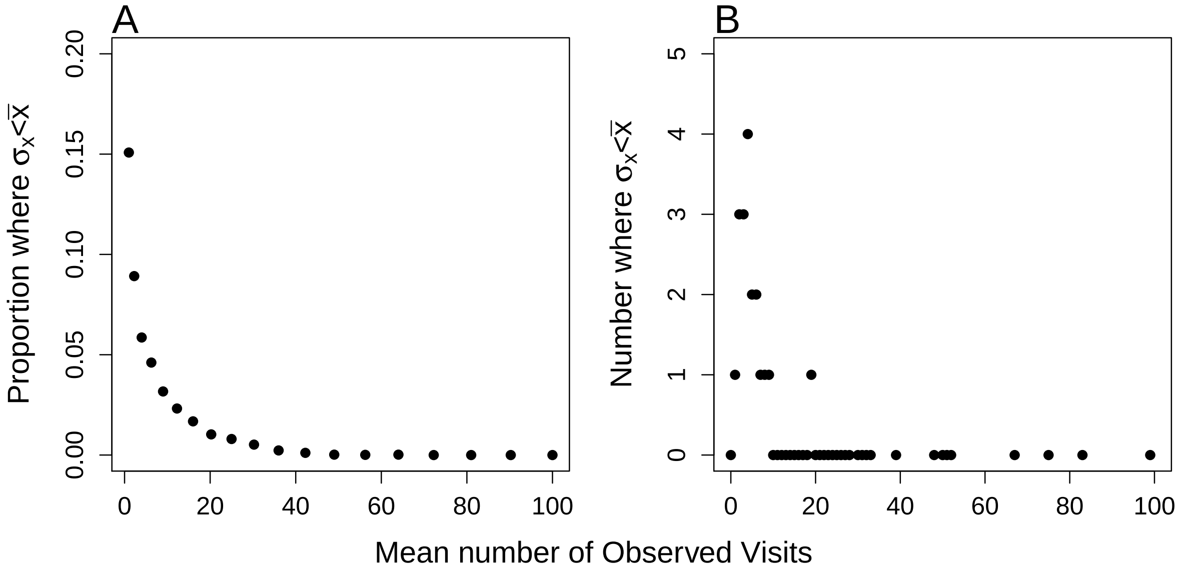

Figure S4: The proportion of samples in which the mean is greater than the variance declines with increasing mean number of observed arrivals, in both A) simulated and B) observed data (from literature review). In B) the mean number of observed arrivals is rounded to the nearest integer. See Supplementary Material S5 for further details of simulations.

S6 Tutorial - Analysing Provisioning Data

Data for this tutorial is included in supplementary files S7, S8 and S9.

S6.1 Exploring Provisioning Rate

Imagine we have collected some provisioning data; we observed 100 nests, each 3 times for 2 hours. Using *R*, lets find out some properties of this provisioning data (`id` is the nest ID, `visits` is the number of visits observed and `time` is the observation time)

```
data_ICC <- read.csv("S7_data_ICC.csv")
head(data_ICC)

##   id visits time
## 1  1     24    2
## 2  1     13    2
## 3  1     24    2
## 4  2     23    2
## 5  2     13    2
## 6  2     27    2

plot(table(data_ICC$visits), xlab="Number of visits", ylab="Frequency", xaxt="n")
axis(1,seq(5,60,5))
```

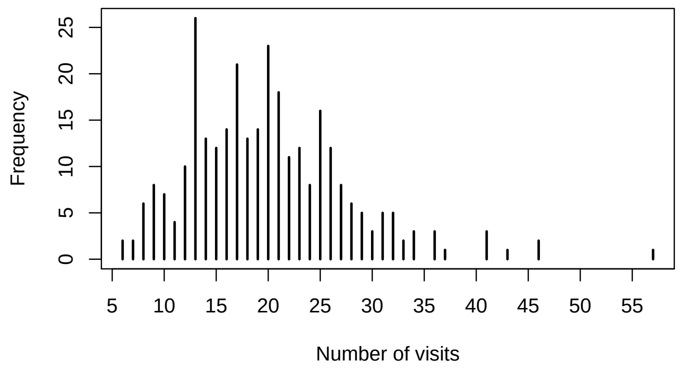

```r
c(mean = mean(data_ICC$visits), variance = var(data_ICC$visits))

##     mean variance
## 19.94333 57.65230
```

We know that the stochastic error is equal to the mean, so we can calculate the variance in expected provisioning rates

```r
var(data_ICC$visits) - mean(data_ICC$visits)

## [1] 37.70896
```

From this we can then calculate the CV of expected provisioning rate

```r
sqrt(var(data_ICC$visits) - mean(data_ICC$visits)) / mean(data_ICC$visits)

## [1] 0.3079105
```

As discussed in Box 2, we may want to present our results as visits/hour; however if we do this, we cannot calculate any of the descriptive statistics mentioned above on this new measure, as we would miscalculate the amount of Poisson-distributed stochastic error. For example if we calculate the expected CV, in this case it is underestimated (and Poisson-distributed stochastic error overestimated).

```r
visits_per_hour <- data_ICC$visits / data_ICC$time
sqrt(var(visits_per_hour) - mean(visits_per_hour)) / mean(visits_per_hour)

## [1] 0.2113453
```

S6.2 Analysing Provisioning Rate as a Response Variable

S6.2.1 Estimating repeatability with and without Poisson-distributed stochastic error

We want to know if the provisioning rate observed at a nest is repeatable. We can run a linear mixed model, as is commonly done in the literature. This assumes no Poisson-distributed stochastic error.

```
## run LMM using lme4 package
library(lme4)
mod_LMM <- lmer(visits ~ 1 + (1|id), data=data_ICC)

## extract ID variance
V_id_LMM <- as.numeric(summary(mod_LMM)$var$id)

## extract residual variance
V_e_LMM <- summary(mod_LMM)$sigma^2

## calculate ICC
ICC_LMM <- V_id_LMM / (V_id_LMM + V_e_LMM)
```

For comparison, we can also run a GLMM, with Poisson error distribution, in order to calculate ICC, which accounts for Poisson-distributed stochastic error. We add in an observation level random effect to calculate over-dispersion variance (Harrison, 2014).

```
## create observation level random effect, i.e a different level for each data point
data_ICC$obs <- 1:nrow(data_ICC)

## run GLMM using lme4 package
mod_GLMM <- glmer(visits ~ 1 + (1|id) + (1|obs), data=data_ICC, family="poisson")
V_id_GLMM <- as.numeric(summary(mod_GLMM)$var$id)
V_e_GLMM <- as.numeric(summary(mod_GLMM)$var$obs)
ICC_GLMM <- V_id_GLMM / (V_id_GLMM + V_e_GLMM)
```

This model can also be run in *MCMCglmm* (Hadfield, 2010), but here we do not have to specify a observation level random effect, as this is done by default. As we get a posterior distribution from *MCMCglmm*, we can calculate the error associated with our ICC estimate.

```
library(MCMCglmm)
mod_MCMCglmm <- MCMCglmm(visits ~ 1, random = ~id, data = data_ICC,
    family = "poisson", verbose = FALSE)
## posterior distribution of individual variance
```

```r
V_id_MCMCglmm <- mod_MCMCglmm$VCV[, "id"]
## posterior distribution of residual variance
V_e_MCMCglmm <- mod_MCMCglmm$VCV[, "units"]
## posterior distribution of ICC
ICC_MCMCglmm <- V_id_MCMCglmm/(V_id_MCMCglmm + V_e_MCMCglmm)
```

Comparing the three, we see that the estimate from the LMM is much lower than when we account for the Poisson-distributed stochastic error in the GLMMs.

```r
cat("LMM =", round(ICC_LMM, 3), "\nGLMM =", round(ICC_GLMM, 3), "\nMCMCglmm =",
    paste0(round(mean(ICC_MCMCglmm), 3), " +/- ", round(sd(ICC_MCMCglmm),
        3)))

## LMM = 0.39
## GLMM = 0.604
## MCMCglmm = 0.607 +/- 0.088
```

Previous work has suggested estimating ICC on the observed rather than expected scale (Nakagawa & Schielzeth, 2010, 2013). This can be done by adding the Poisson-distributed stochastic error back on to the total variance , using the trigamma function (Nakagawa *et al.*, 2017)

```r
## from Nakagawa & Schielzeth 2017
P_error <- trigamma(mean(data_ICC$visits))
V_id_GLMM / (V_id_GLMM + V_e_GLMM + P_error)

## [1] 0.3793103
```

which you can see is similar to what we would predict when not accounting for this stochastic error (i.e. in the LMM). Note this is not what we recommend in this particular case. Conversely, we could 'correct' the ICC estimate from the linear model for the presence of Poisson-distributed stochastic error, by directly removing it

```r
V_id_LMM / (V_id_LMM + V_e_LMM - mean(data_ICC$visits))

## [1] 0.5957898
```

which gives a result similar to that from the Poisson model.

It is common for researchers to log transform this kind of data when analysing it using a LMM, as count data is highly skewed at low means. Repeatability is unaffected by this transformation (here we use log(y+0.5) following Yamamura 1999),

```
mod_LMM_log <- lmer(log(visits + 0.5)~ 1 + (1|id), data_ICC)
V_id_LMM_log <- as.numeric(summary(mod_LMM_log)$var$id)
V_e_LMM_log <- summary(mod_LMM_log)$sigma^2
V_id_LMM_log / (V_id_LMM_log + V_e_LMM_log)

## [1] 0.3736332
```

and Poisson-distributed stochastic error can similarly be corrected for post-hoc using the trigamma function (as it on the log scale).

```
P_error <- trigamma(mean(data_ICC$visits))
V_id_LMM_log / (V_id_LMM_log + V_e_LMM_log - P_error)

## [1] 0.5916202
```

S6.2.2 Estimating CV_B

In a linear mixed model context, CV_B is intuitively calculated as

$$CV_B = \frac{\sqrt{V_B}}{\bar{x}} \tag{S9}$$

, where V_B if the between individual variation (i.e. the individual variance component) (**?**).

```
CV_B_LMM <- sqrt(V_id_LMM) /  mean(data_ICC$visits)
```

This is less simple for non-Gaussian traits. From de Villemereuil *et al.* (2016), we can derive CV_B from a Poisson GLMM as

$$CV_B = \sqrt{V_{B_l}} \tag{S10}$$

, where V_{B_l} is the between individual variance estimated on the link scale (i.e. the individual variance component from the Poisson GLMM). We can compare these values from different models

```
CV_B_GLMM <- sqrt(V_id_GLMM)
CV_B_MCMCglmm <- sqrt(V_id_MCMCglmm)
cat("LMM =", round(CV_B_LMM, 3), "\nGLMM =", round(CV_B_GLMM, 3),
    "\nMCMCglmm =", paste0(round(mean(CV_B_MCMCglmm), 3), " +/- ",
        round(sd(CV_B_MCMCglmm), 3)))

## LMM = 0.238
## GLMM = 0.229
## MCMCglmm = 0.23 +/- 0.026
```

As you can see, CV_B is not affected by the scale (observed or expected) on which it is calculated, as it does not depend on the calculation of total variance.

It is worth noting that if CV_B is estimated using a LMM with log transformed provisioning rate, then CV_B should be calculated as $\sqrt{V_B}$ (again because it is on the log scale).

```
sqrt(V_id_LMM_log)

## [1] 0.2283472
```

S6.2.3 Problems associated with analysing rate

As mentioned in Box 2, some authors have analysed transformed provisioning rate in Poisson model. We presume that authors do this by rounding to the nearest whole number, as most GLMM softwares to not take non-integers as input when a Poisson error distribution is specified.

```
mod_GLMM_rate <- glmer(round(visits_per_hour) ~ 1 + (1 | id) + (1 |
    obs), data = data_ICC, family = "poisson")
V_id_GLMM_rate <- as.numeric(summary(mod_GLMM_rate)$var$id)
V_e_GLMM_rate <- as.numeric(summary(mod_GLMM_rate)$var$obs)
V_id_GLMM_rate/(V_id_GLMM_rate + V_e_GLMM_rate)
```

```
## [1] 1

sqrt(V_id_GLMM_rate)/mean(round(visits_per_hour))

## [1] 0.02269032
```

In this extreme case, the ICC has been estimated as 1 which is clearly a large overestimation, as the model has estimated there being no residual (over-dispersion) variance, whilst CV_B has been hugely underestimated.

S6.2.4 Accounting for observation period

Imagine we observed the same population, but this time we had a large amount of variation in our observation periods.

```
data_ICC_time <- read.csv("S8_data_ICC_time.csv")
head(data_ICC_time)

##   id visits     time
## 1  1     38 4.860166
## 2  1     32 3.847738
## 3  1     39 4.913166
## 4  2     25 2.397478
## 5  2     41 4.788769
## 6  2     32 3.220871
```

Firstly, we can calculate expected CV

```
adj_visits <- (data_ICC_time$visits * mean(data_ICC_time$time))/data_ICC_time$time
sqrt(var(adj_visits) - mean(adj_visits))/mean(adj_visits)

## [1] 0.3218552
```

When estimating ICC, we can correct for this variation in observation period by adding an offset of log observation period. An offset is a covariate where the slope is fixed to 1.

Mathematically, this is equivalent to dividing by the observation period (t), but allows the model to remove the Poisson error before this occurs.

$$log(y) = \beta x + log(t) + \epsilon \tag{S11}$$

$$log(^{y}/_{t}) = \beta x + \epsilon \tag{S12}$$

We can do this with the *lme4* package by using the offset function

```
## create observation level random effect, i.e a different level
## for each data point
data_ICC_time$obs <- 1:nrow(data_ICC_time)
## run GLMM using lme4 package
mod_GLMM_time <- glmer(visits ~ 1 + offset(log(time)) + (1 | id) +
    (1 | obs), data = data_ICC_time, family = "poisson")
V_id_GLMM_time <- as.numeric(summary(mod_GLMM_time)$var$id)
V_e_GLMM_time <- as.numeric(summary(mod_GLMM_time)$var$obs)
ICC_GLMM_time <- V_id_GLMM_time/(V_id_GLMM_time + V_e_GLMM_time)
```

We can also run this in the *MCMCglmm* package, but here the offset function does not work. instead we have to fix the prior of the slope to 1 (or at least make a very informative prior with mean 1 and extremely low variance)

```
## create priors for fixed effects - intercept and obs. time, with
## large variance
prior <- list(B = list(V = diag(2) * 1e+07, mu = c(0, 1)), G = list(G1 = list(V = 1,
    nu = 0.002)))
## make obs. time variance very small
prior$B$V[2, 2] <- 1e-07
mod_MCMCglmm_time <- MCMCglmm(visits ~ log(time), random = ~id, data = data_ICC_time,
    family = "poisson", prior = prior, verbose = FALSE)
V_id_MCMCglmm_time <- mod_MCMCglmm_time$VCV[, "id"]
V_e_MCMCglmm_time <- mod_MCMCglmm_time$VCV[, "units"]
```

```r
ICC_MCMCglmm_time <- V_id_MCMCglmm_time/(V_id_MCMCglmm_time + V_e_MCMCglmm_time)
## compare results
c(GLMM = round(ICC_GLMM_time, 3), MCMCglmm = paste0(round(mean(ICC_MCMCglmm_time),
    3), "+/-", round(sd(ICC_MCMCglmm_time), 3)))
```

```
##            GLMM        MCMCglmm
##         "0.483" "0.485+/-0.074"
```

S6.3 Analysing Provisioning Rate as a Predictor Variable

Imagine we have collected some provisioning data of 100 nests in 2 hour observations. We also collected data on chick mass at the end of the provisioning period.

```r
data_pred <- read.csv("S9_data_pred.csv")
head(data_pred)

##   visits time     mass
## 1     18    2 196.7587
## 2     26    2 181.4664
## 3     17    2 196.4869
## 4     40    2 228.1916
## 5     30    2 222.9193
## 6     25    2 212.5197

par(mfrow=c(1,2))
plot(table(data_pred$visits), xlab="Provisioning Rate", ylab="Frequency", xaxt="n")
axis(1,seq(5,60,5))
hist(data_pred$mass, col="grey", main="", xlab="Chick Mass")
```

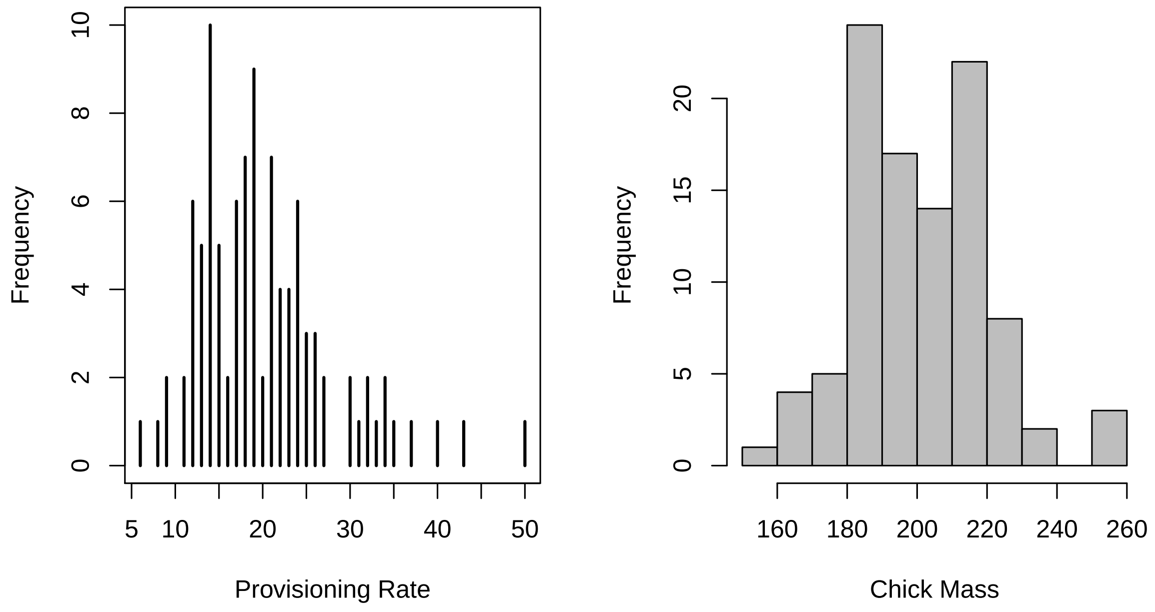

We want to estimate the effect of provisioning rate on chick mass. Traditionally this is done in a linear model

```
mod_LM <- lm(mass~visits, data_pred)
V_f_LM <- var(model.matrix(mod_LM) %*% coef(mod_LM))
V_e_LM <- summary(mod_LM)$sigma^2
R2_LM <- V_f_LM / (V_f_LM + V_e_LM)
R2_LM

##            [,1]
## [1,] 0.4270903
```

This model does not take into account the Poisson-distributed stochastic error in provisioning rate, which leads to an underestimation of effect size. We can account for this using a measurement error model. We can specify such a model in *Stan* (Carpenter *et al.*, 2017) using the following code

```
//modelling uncertainty in a Poisson predictor variable
data {                                    // data being input:
```

```
  int <lower=0> N;                  // number of data points
  vector[N] a;                      // chick body mass
  int <lower=0> v[N] ;              // number_of_visits
}
parameters {                        // parameters to be estimated:
  real beta_0;                      // intercept
  real beta_1;                      // slope of provisioning rate
  real <lower=0> meanPR;            // mean provisioning rate
  real <lower=0> sigmaPR;           // sd provisioning rate
  vector <lower=0> [N] PR;          // provisioning rate
  real <lower=0> sigma;             // residual sd
}
model {
  PR ~ lognormal(meanPR,sigmaPR);
  v ~ poisson(PR);
  a ~ normal(beta_0 + beta_1 * PR,sigma);
}
```

In this code we first tell *Stan* what data we are using, then we define the parameters we want the model to estimate, and then we specify the model and any priors. In essence, what we are doing with this model is creating a latent (unobserved) variable, 'PR', for provisioning rate, and we use this variable in the model for chick mass. For more details about specifying models in *Stan* see http://mc-stan.org.

We can run this model in *R* using the package *rstan*. To do this we can save the stan code in a '.stan' file, and input to *rstan* along with the data, formatted as a list

```
stan_data <- list(N = nrow(data_pred), a = data_pred$mass, v = data_pred$visits)
library(rstan)
## compile the model - this takes a bit of time
stanModel <- stan_model(file = "Poisson_measurement_error_model.stan")
## run the model
```

```r
fit <- sampling(stanModel, data = stan_data, iter = 2000, chains = 1,
    open_progress = F, control = list(max_treedepth = 15, adapt_delta = 0.91))
## extract posterior distirbutions of estimated parameters
ex_fit <- extract(fit, permute = FALSE)
## posterior distributions of latent provisioning rate variable
PR_pred <- ex_fit[, , grep("PR\\[", dimnames(ex_fit)[[3]])]
## posterior distributions of predicted values
outmat <- ex_fit[, , "beta_0"] + ex_fit[, , "beta_1"] * PR_pred[,
    ]
## posterior distribution of fixed effect variance
V_f_stan <- apply(outmat, 1, var)
## posterior distribution of residual variance
V_e_stan <- ex_fit[, , "sigma"]^2
## posterior distribution of R2
R2_stan <- V_f_stan/(V_f_stan + V_e_stan)
```

Comparing the two we can see that that the R^2 from the measurement error model is substantially higher, as the covariance between provisioning rate and chick mass has been better estimated.

```r
c(LM=round(R2_LM,3), stan=paste0(round(mean(R2_stan),3), "+/-", round(sd(R2_stan),3)))

##               LM             stan
##          "0.427" "0.615+/-0.093"
```

S6.3.1 Accounting for observation period

Below is some example code for accounting for observation period when including provisioning rate as a predictor variable. The highlighted code shows the difference with the last code. We have an additional data variable - time (t), and we know that the visits (v) are a function of the provisioning rate (PR) and time, observed with Poisson-distributed stochastic error.

```
//modelling uncertainty in a Poisson predictor variable
```

```
data {                                  // data being input:
  int <lower=0> N;                      // number of data points
  vector[N] a;                          // chick body mass
  int <lower=0> v[N] ;                  // number_of_visits
  vector <lower=0> [N] t;               //observation time
}
parameters {                            // parameters to be estimated:
  real beta_0;                          // intercept
  real beta_1;                          // slope of provisioning rate
  real <lower=0> meanPR;                // mean provisioning rate
  real <lower=0> sigmaPR;               // sd provisioning rate
  vector <lower=0> [N] PR;              // provisioning rate
  real <lower=0> sigma;                 // residual sd
}
model {
  PR ~ lognormal(meanPR,sigmaPR);
  v ~ poisson(t .* PR);
  a ~ normal(beta_0 + beta_1 * PR,sigma);
}
```

References

Bates, D., Mächler, M., Bolker, B. & Walker, S. 2015. Fitting Linear Mixed-Effects Models Using lme4. *J. Stat. Softw.* **67**:1–48.

Carpenter, B., Gelman, A., Hoffman, M.D., Lee, D., Goodrich, B., Betancourt, M. *et al.* 2017. Stan: A Probabilistic Programming Language. *J. Stat. Softw.* **76**:1–32.

de Villemereuil, P., Schielzeth, H., Nakagawa, S. & Morrissey, M. 2016. General methods for evolutionary quantitative genetic inference from generalized mixed models. *Genetics.* **204**:1281–1294.

Hadfield, J.D. 2010. MCMC methods for multi-response generalized linear mixed models: The {MCMCglmm} {R} package. *J. Stat. Softw.* **33**:1–22.

Harrison, X.A. 2014. Using observation-level random effects to model overdispersion in count data in ecology and evolution. *PeerJ.* **2**:e616.

Lendvai, Á.Z., Akçay, Ç., Ouyang, J.Q., Dakin, R., Domalik, A.D., St. John, P.S. *et al.* 2015. Analysis of the optimal duration of behavioral observations based on an automated continuous monitoring system in tree swallows (Tachycineta bicolor): Is one hour good enough? *PLoS ONE* **10**:1–11.

Nakagawa, S., Johnson, P.D. & Schielzeth, H. 2017. The Coefficient of Determination R2 and Intra-Class Correlation Coefficient from Generalized Linear Mixed-Effects Models Revisited and Expanded. *J. Royal Soc. Interface* **14**:20170213.

Nakagawa, S. & Schielzeth, H. 2010. Repeatability for Gaussian and non-Gaussian data: a practical guide for biologists. *Biol. Rev. Camb. Philos. Soc.* **85**:935–56.

Nakagawa, S. & Schielzeth, H. 2013. A general and simple method for obtaining R2 from generalized linear mixed-effects models. *Methods Ecol. Evol.* **4**:133–142.

Yamamura, K. 1999. Transformation using (x + 0.5) to stabilize the variance of populations. *Popul. Ecol.* **41**:229–234.
